# Expansion of the enzymatic repertoire of the CAZy database to integrate auxiliary redox enzymes

**DOI:** 10.1186/1754-6834-6-41

**Published:** 2013-03-21

**Authors:** Anthony Levasseur, Elodie Drula, Vincent Lombard, Pedro M Coutinho, Bernard Henrissat

**Affiliations:** 1 INRA, UMR1163 Biotechnologie des Champignons Filamenteux, Aix-Marseille Université, ESIL Polytech Marseille, 163 avenue de Luminy, CP 925, 13288, Marseille, Cedex 09, France; 2 Architecture et Fonction des Macromolécules Biologiques, UMR6098, CNRS, Aix-Marseille Université, 163 Avenue de Luminy, Marseille, 13288, France

**Keywords:** CAZy database, Evolution of lignocellulose breakdown, Ligninolytic enzymes, Lytic polysaccharide monooxygenases

## Abstract

**Background:**

Since its inception, the carbohydrate-active enzymes database (CAZy; http://www.cazy.org) has described the families of enzymes that cleave or build complex carbohydrates, namely the glycoside hydrolases (GH), the polysaccharide lyases (PL), the carbohydrate esterases (CE), the glycosyltransferases (GT) and their appended non-catalytic carbohydrate-binding modules (CBM). The recent discovery that members of families CBM33 and family GH61 are in fact lytic polysaccharide monooxygenases (LPMO), demands a reclassification of these families into a suitable category.

**Results:**

Because lignin is invariably found together with polysaccharides in the plant cell wall and because lignin fragments are likely to act in concert with (LPMO), we have decided to join the families of lignin degradation enzymes to the LPMO families and launch a new CAZy class that we name “Auxiliary Activities” in order to accommodate a range of enzyme mechanisms and substrates related to lignocellulose conversion. Comparative analyses of these auxiliary activities in 41 fungal genomes reveal a pertinent division of several fungal groups and subgroups combining their phylogenetic origin and their nutritional mode (white vs. brown rot).

**Conclusions:**

The new class introduced in the CAZy database extends the traditional CAZy families, and provides a better coverage of the full extent of the lignocellulose breakdown machinery.

## Background

Decades of research into plant cell wall deconstruction have progressively revealed the enzyme activities involved in lignocellulolysis, for fundamental as well as for applied purposes. Lignin, the main non-carbohydrate structural component of the plant cell wall, is formed of an intricate network of phenolic compounds that constitutes a hard, hydrophobic and insoluble barrier. The plant cell wall carbohydrates form a complex matrix composed of crystalline and insoluble cellulose fibres intimately covered by an intricate network of more easily solubilizable carbohydrate polysaccharides, generically designated as hemicellulose, and, more distantly, by a gel-forming layer of galacturonic acid-containing polysaccharides, commonly designated as pectin. Upon maturation of the cell wall, the phenolic network of lignin is gradually deposited into this matrix and further stabilized through ester bonds to hemicellulose components. Even though the catabolism of each of these individual components can be independently studied, synergy in the breakdown of the various components is commonplace, because the accessibility of each constituent is limited by the presence and nature of the others. Therefore, a global view of the breakdown of all components of the plant cell wall, i.e. cellulose, hemicellulose, pectin and lignin is required to unravel the general mechanisms of lignocellulolysis and to explore the lignocellulolytic capabilities of organisms with sequenced genomes.

The assembly and breakdown of carbohydrate polymers and glycoconjugates are carried out by a diverse panel of Carbohydrate-Active enzymes, or ‘CAZymes’ [1-3]. The CAZymes classification system, based on amino acid sequence similarities, protein folds and enzymatic mechanisms, has been integrated and meticulously updated in the CAZy database (http://www.cazy.org) since 1998. CAZy currently incorporates more than 300 sequence families subdivided into the following classes: glycoside hydrolases (GH), glycosyltransferases (GT), polysaccharide lyases (PL), carbohydrate esterases (CE), and carbohydrate-binding modules (CBM). CAZy provides manually-curated information for each of these categories, making it now possible to cover all known families involved in cellulolysis, hemicellulolysis and pectinolysis.

Lignin forms a three-dimensional network with an aromatic backbone composed of dimethoxylated (syringyl, S), monomethoxylated (guaiacyl, G) and non-methoxylated (*p*-hydroxyphenyl, H) phenylpropanoid units. These phenylpropanoid units are linked by C-C and aryl-ether linkages. The structural complexity, insolubility and high molecular weight of lignin render its enzymatic degradation challenging but rewarding for microorganisms to gain access and convert the polysaccharides. Although lignin degradation is far from being fully deciphered, different microbial enzymatic strategies have already been evidenced to modify and/or degrade the recalcitrant lignin matrix [4-6]. The literature originally opposed two types of plant cell wall-degrading systems: the hydrolytic system responsible for carbohydrate degradation and the oxidative ligninolytic system responsible for lignin depolymerization. Contrary to the subtle specificity of carbohydrate-degrading enzymes, ligninolytic enzymes generate highly-reactive and non-specific free radicals that cleave carbon–carbon and ether inter-unit bonds. Thus, the enzymatic mechanism of ligninolysis has been described as an “enzymatic combustion” involving several non-specific oxidative species. However, such non-specific oxidative action is unlikely to be restricted to lignin digestion and all other plant cell wall components are also susceptible to undergoing oxidation. A number of carbohydrate oxidases have thus been identified in all kingdoms of life, although predominantly in fungi [7]. During microbial attack, these oxidases promote extended cleavage of plant cell wall components and yield reactive molecules (e.g. H_2_O_2_).

Moreover, recent evidence highlights the critical role of alternative enzymatic partners involved in the oxidation of other cell wall components. Originally described as cellulases, GH61 enzymes have recently been shown to be copper-dependent lytic polysaccharide monooxygenases (LPMO) that enhance cellulose degradation in concert with classical cellulases [8-11]. LPMO (GH61) catalyze the oxidative cleavage of cellulose using low molecular weight reducing agents such as ascorbate, gallate, reduced glutathione, and even lignin [12]. It has been also noted that the action of LPMO is strongly potentiated when combined with cellobiose dehydrogenase (CDH) [11,13,14]. Unlike traditional cellulases which require the presence of a loose, isolated substrate chain in the active site cleft or tunnel in order to perform cleavage, the strong oxidative mechanism of LPMO could allow oxidation at the surface of a cellulose crystalline microfibril, where chains are packed against each other. In fact, members of carbohydrate-binding module family CBM33 were first demonstrated to also be oxidative enzymes that cleave polysaccharide chains in crystalline chitin and cellulose [15-17]. The NMR and isothermal titration calorimetry studies of CBM33 demonstrate that these enzymes are also copper-dependent LPMO [18,19]. Therefore, LPMO are essential oxidative enzymes boosting the enzymatic conversion of recalcitrant polysaccharides. As a consequence, today’s modern consensus model for efficient enzymatic decomposition of cellulose should be extended to include not only the synergistic combination of processive and non-processive glycoside hydrolases but also its oxidative cleavage [20].

Cell wall carbohydrates and lignin co-occur in all land plants and are intimately interconnected and covalently cross-linked. We therefore believe that a strict boundary between (hemi)cellulolysis and ligninolysis is perhaps artificial, since functional inference often hinges on the choice of the substrates and analytical methods used in biochemical assays. Moreover, several publications have evidenced a concerted action of specific enzymes able to act on both carbohydrates and lignin [13,21]. In the post-genomic era, where the quest for efficient enzymes for plant cell wall deconstruction has become a major research subject, the simultaneous identification of CAZymes and lignin-degrading enzymes is required to describe the full enzymatic repertoire necessary for plant cell wall deconstruction. Here, we describe the integration of a novel class in the CAZy database. This novel category, broadly termed “Auxiliary Activities” groups together the families of LPMO and the families of redox enzymes involved in lignin breakdown. Like the traditional CAZy families, the new families are based on sequence similarity with one or several biochemically-characterized founding member(s), ensuring that members of a given family share the same three-dimensional structure. Combined with the traditional CAZy families, this addition allows a complete description of the main actors involved in the degradation of all plant cell wall components. Like all enzyme classes in CAZy, the novel class features a hierarchical division in families and, where relevant, clans and subfamilies, all freely accessible at http://www.cazy.org.

## Results and discussion

### Classification of the auxiliary activities

We name “Auxiliary Activities” (AA) a widespread group of catalytic modules involved in plant cell wall degradation. The criteria for integration in the AA classification consists in the potential ability to help the original GH, PL and CE enzymes gain access to the carbohydrates encrusted in the plant cell wall. The AA category therefore encompasses a large class of modules not strictly restricted to a single catalytic reaction mechanism or to specific substrates. All AA families were created according to relevant biochemical characterizations retrieved from the literature for at least one member. The families and lists of AA proteins are continuously updated based on their similarity with the characterized models considered as functional anchors. Within families, subfamilies were manually defined according to their homology relationships between members of the focal family. The newly created AA class now encompasses, improves, replaces and extends a former classification dedicated to fungal ligninolytic enzymes [22]. Moreover, it aims to provide a global list of carbohydrate oxidases. This first release of the current AA class is divided into 10 families, and includes subfamilies for families AA1, AA3 and AA5 (Table 1). Subfamilies are designated using the family name plus a suffix indicating the subfamily. Each AA family and subfamily is concisely described below.

**Table 1:** Subfamily division and known activities in the AA families

**(Sub)families**	**Known activities**	**EC number**	Number of AA^*^
**AA1**	**Multicopper oxidase**		2328
AA1_1	Laccase	EC 1.10.3.2	
AA1_2	Ferroxidase	EC 1.10.3.2	
AA1_3	Laccase-like multicopper oxidase	EC 1.10.3.2	
**AA2**	**Class II peroxidase**		527
	Manganese peroxidase	EC 1.11.1.13	
	Lignin peroxidase	EC 1.11.1.14	
	Versatile peroxidase	EC 1.11.1.16	
**AA3**	**GMC oxidoreductase**		636
AA3_1	Cellobiose dehydrogenase	EC 1.1.99.18	
AA3_2	Aryl-alcohol oxidase / Glucose oxidase	EC 1.1.3.7 / 1.1.3.4	
AA3_3	Alcohol oxidase	EC 1.1.3.13	
AA3_4	Pyranose oxidase	EC 1.1.3.10	
**AA4**	**Vanillyl alcohol oxidase**	EC 1.1.3.38	17
**AA5**	**Radical-copper oxidase**		109
AA5_1	Glyoxal oxidase	EC 1.1.3.-	
AA5_2	Galactose oxidase	EC 1.1.3.9	
**AA6**	**1,4-Benzoquinone reductase**	EC 1.6.5.6	223
**AA7**	**Glucooligosaccharide oxidase**	EC 1.1.3.-	179
**AA8**	**Iron reductase domain**		45
**AA9**	**Lytic polysaccharide monooxygenase (GH61)**	EC 1.-.-.-	249
**AA10**	**Lytic polysaccharide monooxygenase (CBM33)**	EC 1.-.-.-	661

^*^ March 2013.

### Family AA1

The characterized AA1 enzymes are multicopper oxidases that use diphenols and related substances as donors with oxygen as the acceptor. The newly classified AA1 enzymes are widely distributed in a range of organisms from plants, fungi and insects to bacteria. The AA1 family is currently divided into 3 subfamilies as shown below:

Subfamily AA1_1: Laccases (EC 1.10.3.2) are blue copper oxidases that catalyze the one-electron oxidation of phenolics, aromatic amines and other electron-rich substrates *via* the reduction of oxygen to water [23]. These phenol oxidases require the presence of mediator molecules to perform the direct oxidation of high-redox-potential lignin components [24].

Subfamily AA1_2: Ferroxidases, also termed Fe(II):oxygen oxidoreductase, are enzymes present in this subfamily that catalyze the oxidization of iron II to iron III: 4 Fe^2+^ + 4 H^+^ + O_2_ = 4 Fe^3+^ + 2H_2_O. Ferroxidases are key enzymes involved in iron homeostasis and have been extensively studied in the yeast *Saccharomyces cerevisiae* (Fet3p and Fet5p) [25,26]. Through Fe^2+^ oxidation, ferroxidases are potentially able to modulate Fenton reactions or reduce toxic levels of hydroxyl radicals, as suggested in *Phanerochaete chrysosporium*[27].

AA1_3: The laccase-like multicopper oxidases (LMCOs) present here are multicopper oxidases of unknown function. Fungal LMCOs are usually found in Ascomycota, and AA1_3 enzymes are suggested to be involved in diverse functions including lignin degradation, in addition to other physiological roles such as iron metabolism, pathogenic interactions, synthesis of conidial pigments, induction of fruiting bodies, and offensive/defensive fungal strategies [28,29].

### Family AA2

To date, this family contains class II lignin-modifying peroxidases that are part of the plant peroxidase superfamily [30,31]. Initially described as true ligninases due to their high redox potential, class II peroxidases are secreted heme-containing enzymes that use hydrogen peroxide or organic peroxides as electron acceptors to catalyze a number of oxidative reactions in which two electrons are derived from substrate molecules to reduce the enzyme (*via* Compound I and Compound II intermediates) followed by a concomitant release of two water molecules [32]. Known AA2 enzymes include manganese peroxidase (MnP, EC 1.11.1.13), lignin peroxidase (LiP, EC 1.11.1.14) and versatile peroxidase (VP, EC 1.11.1.16). LiPs oxidize phenolic aromatic substrates and catalyze oxidative cleavages of C–C bonds and ether bonds in high-redox-potential non-phenolic aromatic substrates. MnPs oxidize Mn(II) to Mn(III) which in turn oxidize a variety of phenolic model compounds able to degrade and⁄ or modify lignin polymers. VPs are the third type of class II peroxidases included in the family AA2. VPs are the hybrid LIP-like MnP enzymes that combine the catalytic properties of LiP and MnP and are thus able to oxidize Mn(II), phenolic and non-phenolic substrates.

### Family AA3

This family belongs to the glucose-methanol-choline (GMC) oxidoreductases family first outlined by Cavener [33]. AA3 enzymes are flavoproteins containing a flavin-adenine dinucleotide (FAD)-binding domain. Family AA3 can be divided into 4 subfamilies: AA3_1 (mostly covering cellobiose dehydrogenases), AA3_2 (including both aryl alcohol oxidase and glucose 1-oxidase), AA3_3 (alcohol oxidase) and AA3_4 (pyranose 2-oxidase).

Subfamily AA3_1: This subfamily includes cellobiose dehydrogenases (CDH, EC 1.1.99.18) that are extracellular hemoflavoenzymes produced under cellulolytic culture conditions by a number of lignocellulose-degrading fungi [34]. Although direct evidence is still missing, it has been proposed that CDH are involved in cellulose, hemicellulose and lignin biodegradation [35]. CDH employ a wide spectrum of electron acceptors to oxidize soluble cellodextrins to the corresponding lactones [21,36]. They can generate hydroxyl radicals by Fenton-type reactions, thereby potentially oxidizing lignin. In the literature, the core CDH was originally described as a monomeric protein with a bipartite domain organization containing an N-terminal cytochrome domain of spectral class *b* connected to a C-terminal dehydrogenase domain containing the FAD redox cofactor. These two distinct modules in CDH evolved in parallel as fused genes [37]. Therefore, the modularity of CDH has been independently integrated in our classification as two separate modules, i.e. AA3_1 and AA8, respectively. AA3_1 represents the FAD-binding dehydrogenase module also designated cellobiose quinone oxidoreductase CBQ. The modularity of AA enzymes is discussed below.

Subfamily AA3_2: This family includes the two closely-related FAD-dependent enzymes, aryl-alcohol oxidase (AO, EC 1.1.3.7) and glucose 1-oxidase (GOX, EC 1.1.3.4). In our sequence-based classification, AO and GOX are strongly related and found in the same subfamily AA3_2. AO enzymes catalyze the oxidative dehydrogenation of several aromatic and aliphatic polyunsaturated alcohols with an α-carbon primary hydroxyl group, with the concomitant reduction of O_2_ to H_2_O_2_. GOX oxidizes the hydroxyl group at the C1 position of sugars. Both AO and GOX are H_2_O_2_-generating enzymes required for class II peroxidases to oxidize lignin. Lignin oxidation can alternatively be driven by reduction of H_2_O_2_ to hydroxyl free radical (OH^•^) via ferrous iron recycling [38,39]. GOX-generated H_2_O_2_ (intracellular enzymes) requires an export system that has not been reported to date [40]. In addition, related and more distant GMC oxidoreductases have been added in this subfamily based on sequence similarity to AO and GOX.

Subfamily AA3_3: Thus far, this subfamily contains members characterized as alcohol (methanol) oxidases (AlcOx; EC 1.1.3.13). AlcOx catalyze the oxidation of primary aliphatic alcohols to their respective aldehydes. They are able to use the methanol produced during lignin attack (demethoxylation) to supply the H_2_O_2_ necessary for hydroxyl radical generation in Fenton reactions [41].

Subfamily AA3_4: To date the only known activity in this subfamily is pyranose 2-oxidases (P2O, EC 1.1.3.10), which catalyze the C2-oxidation of D-glucopyranose and structurally analogous mono- and di-saccharides to the corresponding sugars while reducing O_2_ to H_2_O_2_[42].

### Family AA4

The characterized members of this family are exclusively vanillyl-alcohol oxidases (VAO) that catalyze the conversion of a wide range of phenolic compounds bearing side chains at the *para*-position of the aromatic ring [43]. A broad spectrum of aromatic compounds is produced during the degradation of lignin, and AA4 enzymes are known to be active with a wide variety of these key intermediates.

### Family AA5

The AA5 family belongs to the copper radical oxidases family and includes two subfamilies, namely AA5_1 containing characterized glyoxal oxidases and AA5_2 containing galactose oxidases. AA5_1 and AA5_2 share similar tertiary structures and virtually identical active sites despite different catalytic specificities and low sequence similarity.

Subfamily AA5_1: Glyoxal oxidases are copper-radical oxidases with a broad specificity able to oxidize simple aldehydes to the corresponding carboxylic acids [44]. Glyoxal oxidases are considered one of the central H_2_O_2_-generating enzymes. AA5_1 also integrates glyoxal-like sequences called cro1 to cro6 genes that share low overall sequence similarity to glyoxal oxidase but have conserved residues surrounding the catalytic site [45].

Subfamily AA5_2: Galactose oxidases (GAO) present here are copper-containing enzymes that catalyze a reaction comprising two separable half-reactions, oxidation of a primary alcohol, and reduction of O_2_ to H_2_O_2_[46]. GAO catalyze the oxidation of a wide range of carbohydrates (including galactose) but also primary alcohols into the corresponding aldehydes, with the reduction of O_2_ into H_2_O_2_.

### Family AA6

This family contains members among which all experimentally characterized proteins are 1,4-benzoquinone reductases. These are intracellular enzymes involved in the biodegradation of aromatic compounds and in the protection of fungal cells from reactive quinone compounds. During the fungal degradation of aromatic compounds, quinones are key intermediate metabolites. Benzoquinone reductases are involved in a quinone redox cycle that generates extracellular Fenton reagents [47-49].

### Family AA7

The gluco-oligosaccharide oxidases (GOO) found in this family oxidize the C1 hydroxyl groups of *β*-1,4-linked sugars through two half-reactions featuring the oxidation of the reducing sugar to the lactone and spontaneous hydrolysis to the corresponding acid [7]. GOO are able to oxidize a variety of carbohydrates (D-glucose, maltose, lactose, cellobiose, malto- and cello-oligosaccharides) with the concomitant reduction of O_2_ to H_2_O_2_[50]. Known AA7 enzymes are potentially involved in the biotransformation or detoxification of lignocellulosic compounds.

### Family AA8

Iron reductases consist in a cytochrome domain (protoheme IX) of spectral class *b*. Iron reductase was first described as the N-terminal hemic module found in the bipartite domain organization of the flavocytochrome CDH. In *P. chrysosporium*, AA8 stimulates the reduction of Fe(III) by acting as an electron sink or by directly reducing Fe(III) [51]. The physiological role of this family is unclear, but it could participate in the generation of highly reactive hydroxyl radicals (OH^•^) via Fenton’s reaction. Consequently, AA8 proteins could be involved in the non-enzymatic cellulose chain breakage that involves hydroxyl radical attack [52]. Moreover, the C-terminal CBM1 might bring the generation of hydroxyl radicals by the Fenton’s reaction in the vicinity of cellulose.

### Family AA9

This family includes the lytic polysaccharide monooxygenases previously classified as family GH61 glycoside hydrolases [8,53]. AA9 enzymes have been recently characterized as copper-dependent polysaccharide monooxygenases containing highly conserved histidine residues implicated in a type-2 copper center. These enzymes are believed to act directly on cellulose, rendering it more accessible to more traditional CAZyme action [16]. The oxidative cleavages of cellulose catalysed by AA9 enzymes require reducing equivalents generated by the AA3_1 enzyme or alternatively low-molecular-weight reducing agents [8,12]. AA9 enzymes are shown to oxidize the C1 carbon of the glucose ring structure but may also be able to oxidize C4 and C6 [9,11,14]. A number of AA9 members have been identified mainly in fungal genomes, indicating the widespread nature of this family, especially in the fungal wood decayers.

### Family AA10

AA10 renames carbohydrate-binding module family CBM33. As for family AA9, the newly renamed AA10 members are copper-dependent monooxygenases that utilize molecular oxygen and external electron donors [18,19]. Known AA10 enzymes are capable of oxidatively cleaving polysaccharide chains in crystalline chitin and cellulose to produce aldonic acids [15,17]. AA10 action should increase the substrate accessibility for hydrolytic enzymes, and thus act synergistically with chitinases and cellulases. AA10 are structurally similar to AA9. Both families are devoid of classic substrate binding grooves or pockets yet they possess distinctive flat substrate-binding surfaces. Family AA10 also includes a conserved N-terminal histidine residue which is an integral component of the metal binding site. AA10 proteins are not limited to a specific kingdom, but are so far predominantly represented in bacteria [54].

### Evolutionary relationships and modularity in AA families

**Figure 1:**
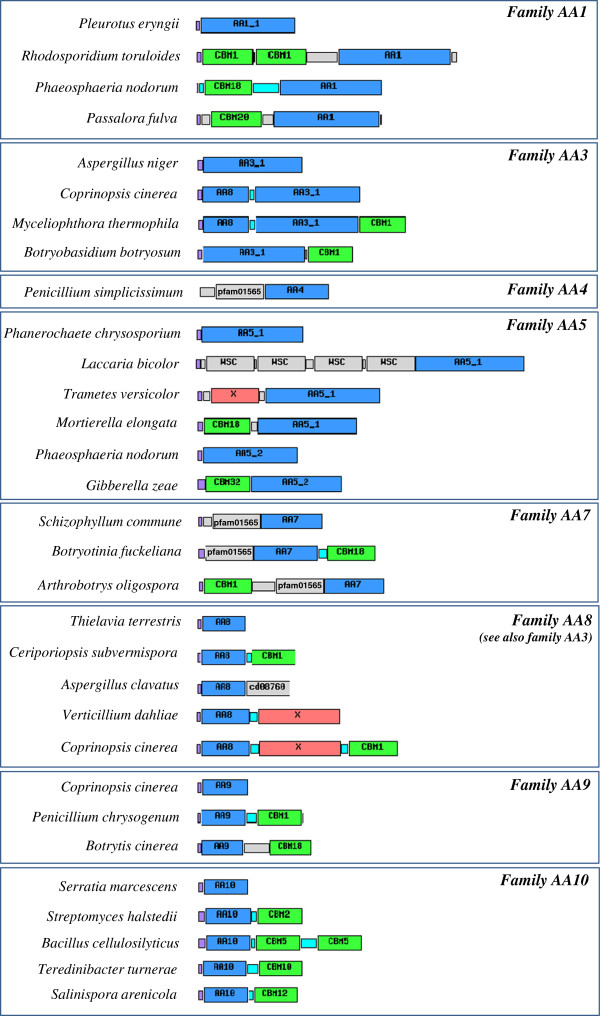
**Examples of modular AA proteins.** The additional modules represented include: carbohydrate-binding modules (CBMs; green), conserved modules of unknown function (X; red) and other domains (labelled grey boxes). Signal peptides (purple), proline/serine-rich linkers (cyan) and unassigned connecting regions (unlabelled grey boxes) are also shown.

Over the course of evolution, fungal genomes have been shaped by a series of complex processes leading to changes in biochemical function that are reflected in the amino acid sequence [55-59]. Sequences evolve as a continuum in which positive, neutral and purifying selection drive the pace of the fixed mutations. Furthermore, new protein architectures (module gains, losses and shuffling) are frequently observed to occur and are thus recurrent evolutionary events shaping a given protein. As a rule, ligninolytic enzymes were found to be less modular than cellulolytic enzymes suggesting that close contact with polysaccharides is less compulsory. Notable exceptions were made for certain AA families in which various modular combinations were clearly evidenced (Figure 1). As recurrently observed in the established CAZy families, AA families have multimodular structures composed of catalytic modules associated to different ancillary modules like CBMs. This modular distribution is not uniformly shared across all AA families but remains strongly family-dependent. Families AA1, AA3, AA4, AA5, AA7, AA8, AA9 and AA10 are especially prone to modular organization.

The AA1 family was surprisingly found to be associated to various CBMs (CBM1, CBM18 and CBM20), which is singular for multicopper oxidases. In agreement with the diverse functions attributed to the AA1 family, the modular multicopper oxidases may be functionally linked to the modification of unexpected substrates such as chitin or even starch. Among the AA3 subfamilies, AA3_1 possesses a modular organization where the respective modules can be found either isolated or associated to the N-terminal cytochrome module AA8 and/or to a C-terminal CBM1. Despite different possible associations between AA8, AA3_1 and CBM1, their modular arrangement is strictly conserved. The glyoxal and glyoxal-like modules included in subfamily AA5_1 can contain one or more N-terminal WSC modules in tandem with the catalytic copper radical oxidase module. WSC domains are approx. 100 amino acid long with eight conserved cysteines, which are predicted to form disulfide bridges. The function of WSC domains is not known. Concerning subfamily AA5_2, N-terminal CBM13 and CBM32 modules can be found associated to the core catalytic module. A similar association was identified in AA7 in which associations to CBM12 and CBM18 modules are recurrent, which could point to a potential role of AA7 in the chitin modification. Interestingly, a shared modular organization was also evidenced between the AA4 and AA7 families that feature an N-terminal FAD-binding-4 module (pfam01565). The common modular organization between AA4 and AA7 reflects their shared ability to use FAD as a co-factor. As described above, AA8 is frequently associated to AA3_1, but AA8 can also be found fused to distinct modules. In fact, AA8 can be produced either as a single module or associated to AA3_1, CBM or other unknown modules. It is therefore possible that AA8 could be used as a key functional partner for the discovery of new candidate modules potentially involved in plant cell wall degradation.

Concerning the LPMO families AA9 and AA10 (formerly GH61 and CBM33, respectively), both are frequently multimodular and can bear a C-terminal CBM. To date, AA9 have only been found in association with CBM1 and CBM18, whereas AA10 are frequently fused to one or several modules of family CBM2, CBM5, CBM10, CBM12, and fibronectin type-III modules. Interestingly, the AA9 and AA10 enzymes always bear their ancilliary modules at the C-terminus, in agreement with the critical role of the N-terminal histidine for enzyme activity. Indeed, the first N-terminal histidine is involved in the coordination of the copper ion and the loss of this position would render the protein inactive.

In conclusion, diverse modular organizations are frequently detected in the AA families. The evolution of ligninolytic oxidoreductases makes use of classical evolutionary events in which shuffling events make it possible to associate different modules together. Gain and loss of modularity in AA families are both subsequently driven by common selective mechanisms (conservative, neutral or Darwinian selection), leading to different fates for “neo”-modular emergences. The modularity of ligninolytic modules has historically been underestimated. Additional modular architectures will doubtless be discovered in the near future by exploring novel and original taxonomic groups via systematic genome sequencing.

### Occurrence of AA genes in fungal genomes

**Figure 2:**
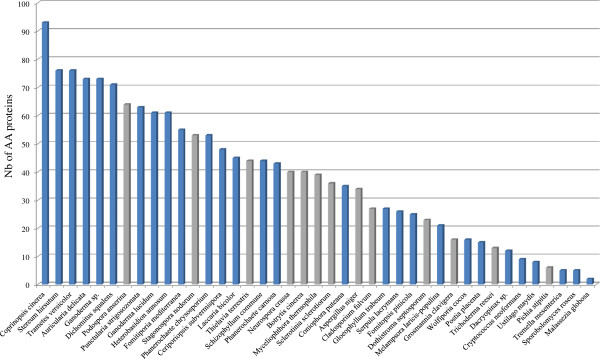
**Number of AA proteins in the 41 selected fungal genomes.** Basidiomycetes are depicted in blue and ascomycetes are depicted in grey.

**Figure 3:**
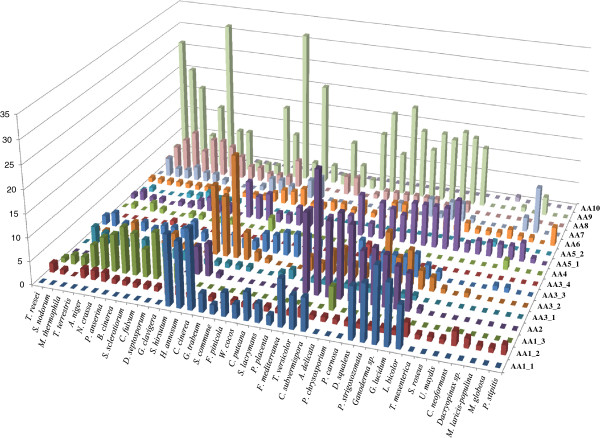
Distribution of AA proteins in the 41 selected fungal genomes.

We selected 41 fungal genomes in order to compare their AA gene contents. The selected genomes mainly included several well-known white rot fungi (WR fungi), brown rot fungi (BR fungi) and plant pathogens from the Ascomycota (13 species) and Basidiomycota (28 species) divisions. A total of 1576 AA were identified in the 41 genomes. The AA distribution was not uniform across species (Figure 2). *Coprinopsis cinerea*, *Stereum hirsutum* and *Trametes versicolor* possess the highest number of AA (> 75 putative AA) while *Malassezia globosa*, *Sporobolomyces roseus* and *Tremella mesenterica* possess the lowest number of AA (≤ 5 putative AA). The well-known “industrial fungus” *Trichoderma reesei* is also particularly poor in AA enzymes as also demonstrated for the traditional CAZy families [60]. The distribution of the AA families was compared for each fungal genome, demonstrating that different enzymatic tactics are employed across the fungal kingdom (Figure 3, Additional files 1 and 2).

**Figure 4:**
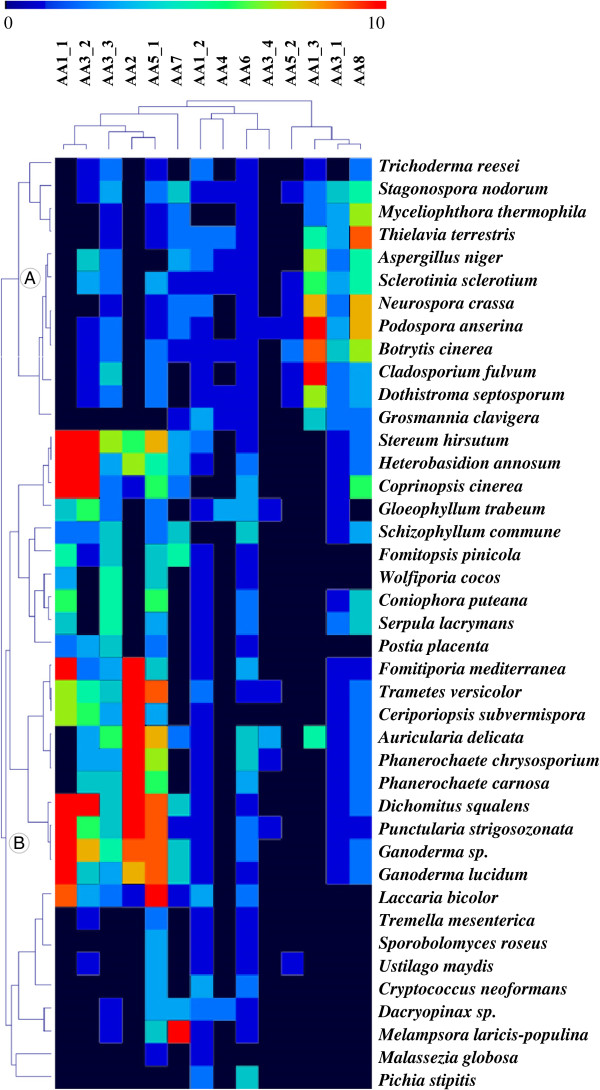
**Comparison of the ligninolytic AA repertoires identified in the selected fungal genomes using double hierarchical clustering.** Top tree: family number according to the AA classification. Left tree: Fungal genomes analyzed. **A** and **B** correspond to the Ascomycotina and Basidiomycotina divisions, respectively. The abundance of the different AA proteins within a family is represented by a colour scale from 0 (dark blue) to ≥ 10 occurrences (red) per species. Note that only ligninolytic AA families were selected (AA1 to AA8).

In order to organize the dataset into meaningful groups and identify coherent patterns, a hierarchical clustering of the number of ligninolytic AA family members was generated. Only ligninolytic families AA1 to AA8 were considered to focus exclusively on the lignin-degrading abilities of the 41 fungi including some important WR and BR representatives. Clustering of the AA dataset identified several major groups and subgroups of species that are discussed below according to phylogenetic origin and nutritional mode (Figure 4, Additional file 3).

#### Phylogenetic origin

Two main groups appeared which corresponded to the Ascomycota and Basidiomycota phyla. In evolution, Ascomycota and Basidiomycota emerged around ~520 Myr after the split of the common ancestor (Dikarya) [61], with each group evolving its own set of ligninolytic enzymes. Ascomycota genomes contained no identifiable class II peroxidases (AA2) or ligninolytic laccase (AA1_1) whereas all Ascomycota possessed laccase-like multicopper oxidases (AA1_3). Surprisingly, AA1_3 (but not AA1_1) was also observed in the basidiomycete *Auricularia delicata*. This inconsistency between the current phylogeny including AA1_3 and the traditionally-accepted phylogeny of *A. delicata* poses a number of questions. *A. delicata* is one of the earliest branching Agaricomycete lineages, meaning that AA1_3 could either have been inherited and conserved from a common ancestor or acquired by horizontal transfer [62]. Ascomycota possess a higher number of AA3_1 (CDH) than Basidiomycota (averaging ~2.3 *vs* ~0.6, respectively). Only AA3_1 from Ascomycota could be fused to a C-terminal CBM1 module (~28% of total AA3_1) [34,63]. The acquisition of a CBM1 may have occurred early before the diversification of the AA3_1 family in the Ascomycota division, or alternatively this modular organization may have been prematurely lost in Basidiomycota. As expected, Ascomycota are particularly prone to a high proportion of AA8 family members compared to Basidiomycota (~5 copies/genome *vs* ~1.5 copy/genome, respectively). Interestingly, AA8 can be individually fused to CBM1 at the C-terminal end (only), and this modular organization occurred in both fungal divisions. In terms of AA content, the non-ligninolytic *Pichia stipitis* stands out as containing only a few AA and clustering with low-AA-content genomes such as *M. globosa*. *P. stipitis* belongs to the subphylum Saccharomycotina, contrary to the other fungal Ascomycota genomes which belong to the subphylum Pezizomycotina. Basidiomycota presented significant differences in AA family distribution compared to Ascomycota. Subfamily AA1_1 ligninolytic laccases, like AA2 class II peroxidases, were only identified in Basidiomycota. AA5_1 was prevalent in Basidiomycota with a mean of ~5 copies per genome *vs* ~1 copy in Ascomycota.

#### Nutritional mode

Among the Basidiomycota sequenced to date, all BR fungi lack ligninolytic AA2 enzymes whereas WR fungi contain up to 26 AA2 proteins and average ~11 AA2 copies per genome. This high prevalence of AA2 enzymes in WR fungi re-enforced the central role of ligninolytic class II peroxidases in wood degradation, and argues for the use of AA2 as a family “marker” for predicting the wood-decaying activities and nutritional mode of different fungal groups (WR *vs* BR). Also worthy of mention is that known BR fungi such as *F. pinicola*, *W. cocos* and *P. placenta* possess “low redox potential peroxidases” lacking residues involved in Mn^2+^ binding. These “low redox potential peroxidases” may oxidize low redox-potential dyes and phenols, but their putative involvement in plant cell wall degradation needs to be experimentally studied before drawing more conclusions (Additional file 2) [64]. No AA2 enzyme was found in *S. commune* despite the fact that it was initially considered a WR fungus although its lignin-degrading capacity is limited [65]. Based on this similarity with BR fungi, *S. commune* clustered into the BR group. Concerning the AA3_2 family (AO and GOO), fewer enzymes were comparatively found among BR fungi, especially in *F. pinicola*, *W. cocos*, *C. puteana*, *S. lacrymans* and *Dacryopinax sp*. In WR fungi, AO enzymes are known to participate in the fungal degradation of lignin by providing the H_2_O_2_ required by ligninolytic peroxidases. This result is in agreement with the high AA2 content in WR fungi. Although subfamily AA3_1 was considered an important actor of lignocellulolysis [66], we found no significant correlation with the nutritional modes. However, the AA3_1 subfamily is prevalent in some “soft rot fungi” from the Ascomycota group, along with the AA1_3 subfamily.

*Dacryopinax sp*. (“jelly fungi” belonging to the Dacrymycetales order) has a limited number of AA families, including no member of subfamily AA1_1 among BR, and was clustered apart from other BR fungi. Based on the distribution of fungal species, the emergence of BR fungi seems to have followed a different evolutionary scenario. BR fungi may have appeared several times in evolution, accompanied by reductions in specific families. The chronology of divergences in fungal nutritional modes and the origin of lignin degradation were recently deciphered using comparative and functional genomics [52,61]. BR fungi are able to perform an initial nonenzymatic attack on the wood cell wall, generating hydroxyl radicals (OH^•^) extracellularly *via* the Fenton reaction. The generation of H_2_O_2_ could be achieved by oxidases, especially the high number of AA5_1 copper radical oxidases present in BR fungi (averaging ~3.4 copies per genome). In WR fungi, the species *A. delicata*, *P. carnosa* and *P. chrysosporium* belong to a separate cluster in which no subfamily AA1_1 was found. Although distant multicopper oxidases could be identified in these three WR species genomes, the absence of classical ligninolytic laccases revealed different enzymatic panels and, subsequently, different evolutionary groups within WR-mode lineages. Although the ability of laccases to remove lignin has been demonstrated during delignification [67], AA1_1 ligninolytic laccases are not compulsory *sensu stricto* in the WR mode of wood decay.

### Co-occurrence of AA families in fungal genomes

In addition to identifying clusters of species, the AA families clustered into groups of biological significance (Figure 4). For instance, AA1_1, AA3_2, AA3_3, AA2 and AA5_1 clustered together. The AA2-family class II peroxidases require H_2_O_2_, and several extracellular enzymes have been postulated to be involved in extracellular H_2_O_2_ production. As expected, the main H_2_O_2_-producers included members of the families AA5_1 (known for glyoxal oxidase activity), AA3_2 (including AO and GOX) and AA3_3 (known as AlcOx). Interestingly, AA1_1 enzymes may also be involved synergistically with AA3_2 members to produce hydroxyl radicals, as suggested in this cluster. Indeed, laccases are able to produce hydroxyl radical (OH^•^) during the oxidation of hydroquinones, and the addition of AO from subfamily AA3_2 strongly increases OH^•^ generation [68]. AA1_3, AA8 and AA1_3 also clustered together. AA8 and AA3_1 share a modular organization that means they are preferentially identified together. The laccase-like multicopper oxidases AA1_3 were grouped with the AA8 and AA3_1 families essentially based on the fact that all three families are found in high contents in Ascomycota. Interestingly, there is already evidence of synergistic action between these families, as it was demonstrated that the combined action of CDH and laccases resulted in substantial increases in the decolorization of synthetic dyes [69-71].

### Multigenicity in the AA families

The annotation of AA in the fungal genomes revealed a striking discrepancy between the numbers of members within families. The multiplicity of members mainly concerns families AA1_1, AA2, AA3_2 and AA9 in which up to 17, 26, 22 and 33 candidate proteins were assigned in individual genomes, respectively (Additional file 1). In contrast, other families seem particularly stable or less populated (i.e. AA3_1 ranged from 0 to 4 AA per genome). Interestingly, family expansions occurred in both ligninolytic AA (sub)families (AA1_1, AA2) and in AA9 which are likely involved in the oxidation of polysaccharides. From an evolutionary standpoint, the expansion of specific families is particularly intriguing. Family expansions are not randomly distributed, as only specific families are repeatedly targeted in several genomes, which is synonymous with convergence in evolution. A driving force for the emergence of multigenic families and gene novelty is the duplication event [72]. After duplication, the probability of fixation and maintenance of duplicates depends on numerous variables, and a combination of stochastic but also adaptive mechanisms take place. The remarkable expansion of the AA1_1, AA2 and AA9 families raises the question of the selective pressures constraining these families, and consequently the functional relevance at the organismal level (i.e. fitness). Different scenarios can be hypothesized to explain the multigenic AA families, ranging from i) functional redundancy to ii) functional diversification or iii) fine-tuned regulation of alternative genes. As fungi have to contend with the high complexity of the plant cell wall, the expansion of the AA families may reflect adaptations to the degradation of the substrates or to the use of different cofactors present *in situ* in the plant cell wall.

## Conclusion

Plant cell wall degradation requires an arsenal of catalytic and non-catalytic proteins produced by lignocellulolytic microorganisms. Lignocellulolysis is a complex process, and fungi have evolved different enzymatic strategies to utilize these substrates as carbon sources. The growing number of published genome sequences and the allied “omics” data offers a unique opportunity for gaining deeper insight into the evolution of lignocellulolysis and the families involved in this biological machinery of high biotechnological potential. The novel AA classification provides a complementary insight into enzymatic lignocellulolysis by focusing on oxidative enzyme families. All known lignocellulolytic categories are now present in the continuously updated CAZy database. This expansion of the scope of the database is not restricted to the breakdown of plant cell wall as family AA10 (formerly CBM33) has been shown to act not only on cellulose, but also on another recalcitrant and crystalline polysaccharide, chitin [15-17]. The presently released AA families are strongly biased towards the fungal kingdom. New kingdoms will be explored in the near future, especially in Bacteria where lignin breakdown may be more significant than previously thought [73]. Indeed, there are a number of literature reports of soil bacteria or aromatic-degrading bacteria from the digestive microbiota of animals that are able to depolymerise lignin (e.g. *Nocardia*, *Rhodococcus*, *Sphingobium; Streptomyces*) [74-76]. The precise enzymology of bacterial lignin conversion is still in infancy and enzyme families involved in lignin breakdown will be added to the AA category in due time. It is hoped that the common annotation procedures and the dissemination provided by the CAZy database framework will allow cross fertilization of the various components of lignocellulose research. Rational integration of experimentally determined functional data as described earlier for traditional CAZymes [3] allied to biochemical expertise, will ultimately make it possible to provide a reliable and evolving functional annotation of all enzymes involved in the breakdown of lignocellulose and other recalcitrant biopolymers.

## Methods

### Family creation and propagation

The new families were created based on sequence similarity criteria analogous to those used in the past to define CAZy families [3]. Family creation starts with the identification of biochemically-characterized enzyme sequences that are decomposed into functional modules using available structural data whenever possible, followed by comparisons and manual curation. The modularity of these seeding examples is propagated to clearly identify homologous sequences using BLASTP [77] against GenBank sequences [78] in the initial stages of family definition. Families GH61 and CBM33 were just renamed (respectively AA9 and AA10) and proper information has been added to the headers of former families GH61 and CBM33 to indicate this fact. It is important to stress that inclusion of new sequences in the families is driven solely by sequence similarity and not by taxonomy or biochemistry considerations. In consequence, some families are obviously multi-functional and these were decomposed into subfamilies using criteria similar to those used in prior subfamily analyses [79-81].

### Hierarchical clustering

The number of each representative for each ligninolytic family (AA1 to AA8) was listed in order to compare the ligninolytic abilities of the 41 selected fungi. Data were ordered and grouped using the distance matrix computation and hierarchical clustering analysis calculated by MultiExperimentViewer [82]. A double clustering was carried out based on Pearson correlation as a distance matrix and complete linkage clustering as a linkage method.

## Abbreviations

AA: Auxiliary Activities; CAZy: Carbohydrate-Active enZymes; AlcOx: Alcohol oxidase; AO: Aryl-alcohol oxidase; BR: Brown rot; CBM: Carbohydrate-binding modules; CDH: Cellobiose dehydrogenase; CE: Carbohydrate esterase; GAO: Galactose oxidase; GH: Glycoside hydrolase; GMC oxidoreductase: glucose-methanol-choline oxidoreductase; GOO: Gluco-oligosaccharide oxidase; GOX: Glucose-1-oxidase; GT: Glycosyltransferases; LMCO: Laccase-like multicopper oxidase; LPMO: Lytic polysaccharide monooxygenase; Myr: Million years; PL: Polysaccharide lyase; MnP: Manganese peroxidase; LiP: Lignin peroxidase; VAO: Vanillyl-alcohol oxidase; VP: Versatile peroxidase; WR: White rot

## Competing interests

The authors declare that they have no competing interests.

## Authors’ contributions

AL and BH conceived the study. AL designed and carried out the bioinformatics studies. ED and VL contributed to the data integration into the database. PMC helped to conceive the study. AL drafted the manuscript. AL and BH wrote the final manuscript. All authors have read and approved the final manuscript for publication.

## Supplementary Material

Additional file 1 Number of AA proteins in the 41 selected fungal genomes. Click here for file

Additional file 2 List of AA proteins identified in the 41 selected fungal genomes. Click here for file

Additional file 3: **Schematic distribution of ligninolytic AA in fungal genomes.** Each genome is represented as a circle. The total number of AA is given in the middle of each circle. Groups 1 to 4 were defined according to the hierarchical clustering. (PDF 7277 kb) Click here for file
