# Supplementary material for: Expansion of the enzymatic repertoire of the CAZy database to integrate auxiliary redox enzymes
Source: Biotechnol Biofuels. 2013 Mar 21;6:41. doi: 10.1186/1754-6834-6-41 (PMC3620520; doi:10.1186/1754-6834-6-41)
Supplement: Additional file 1 — Number of AA proteins in the 41 selected fungal genomes. [file 1754-6834-6-41-S1.pdf]

Supplemental data S1. Number of AA proteins in the 41 selected fungal genomes.

	AA1_1	AA1_2	AA1_3	AA2	AA3_1	AA3_2 [a]	AA3_3	AA3_4	AA4	AA5_1	AA5_2	AA6	AA7	AA8	AA9	AA10
Aspergillus niger	0	2	7	0	2	4	2	0	1	0	0	1	3	5	7	0
Auricularia delicata	0	1	5	18	1	3	6	3	0	8	0	4	2	2	20	0
Botrytis cinerea	0	1	9	0	4	1	2	0	1	2	2	1	1	7	9	0
Ceriporiopsis subvermispora	7	1	0	16	1	6	3	0	0	3	0	0	0	2	9	0
Cladosporium fulvum	0	1	10	0	2	1	4	0	0	2	1	1	0	3	2	0
Coniophora puteana	6	1	0	0	1	0	5	0	0	6	0	2	0	4	10	0
Coprinopsis cinerea	17	0	0	1	1	22	2	0	0	6	0	3	2	6	33	0
Cryptococcus neoformans	0	3	0	0	0	0	0	0	0	3	0	2	0	0	1	0
Dacryopinax sp.	0	2	0	0	0	0	1	0	2	3	0	1	3	0	0	0
Dichomitus squalens	11	1	0	12	1	11	4	0	0	9	0	1	4	2	15	0
Dothistroma septosporum	0	1	7	0	2	1	2	0	1	2	1	1	0	3	2	0
Fomitiporia mediterranea	10	1	0	17	1	2	3	0	0	4	0	3	0	1	13	0
Fomitopsis pinicola	5	1	0	0	0	1	4	0	0	4	0	1	5	0	4	0
Ganoderma lucidum	13	1	0	8	1	4	3	0	0	9	0	1	4	2	15	0
Ganoderma sp.	16	1	0	9	1	8	5	0	0	9	0	2	4	2	16	0
Gloeophyllum trabeum	4	1	0	0	1	6	2	1	3	2	0	3	0	0	4	0
Grosmannia clavigera	0	3	4	0	2	0	0	0	1	0	0	1	1	2	2	0
Heterobasidion annosum	14	1	0	7	1	13	3	0	0	5	0	2	3	2	10	0
Laccaria bicolor	9	3	0	1	0	3	2	0	0	11	0	2	1	0	13	0
Malassezia globosa	0	1	0	0	0	0	0	0	0	1	0	0	0	0	0	0
Melampsora laricis-populina	0	1	0	0	0	0	1	0	0	4	0	1	10	0	4	0
Myceliophthora thermophila	0	0	2	0	3	0	1	0	0	1	0	1	2	7	22	0
Neurospora crassa	0	2	8	0	2	0	1	0	0	1	1	1	2	8	14	0
Phanerochaete carnosa	0	1	0	11	1	4	4	0	0	6	0	3	0	2	11	0
Phanerochaete chrysosporium	0	1	0	16	1	3	3	1	0	7	0	4	0	2	15	0

Pichia stipitis	0	2	0	0	0	0	0	0	0	0	0	4	0	0	0	0
Podospora anserina	0	1	10	0	3	1	2	1	0	1	1	1	2	8	33	0
Postia placenta	2	1	0	0	0	3	4	0	0	2	0	1	0	0	2	0
Punctularia strigosozonata	12	1	0	11	1	6	4	1	0	9	0	2	1	1	14	0
Schizophyllum commune	2	0	0	0	1	2	4	0	0	2	0	4	4	3	22	0
Sclerotinia sclerotiorum	0	1	6	0	3	3	2	0	1	3	1	1	1	5	9	0
Serpula lacrymans	4	1	0	0	2	0	5	0	0	3	0	2	0	4	5	0
Sporobolomyces roseus	0	1	0	0	0	0	0	0	0	3	0	1	0	0	0	0
Stagonospora nodorum	0	1	2	0	4	1	3	0	1	2	1	1	4	5	28	0
Stereum hirsutum	15	2	0	6	1	15	7	0	0	8	0	1	3	2	16	0
Thielavia terrestris	0	2	5	0	3	0	1	0	2	1	0	1	2	9	18	0
Trametes versicolor	7	2	0	26	1	5	4	1	0	9	0	1	0	2	18	0
Tremella mesenterica	0	1	0	0	0	1	0	0	0	2	0	1	0	0	0	0
Trichoderma reesei	0	2	1	0	0	1	2	0	0	1	0	1	0	2	3	0
Ustilago maydis	0	1	0	0	0	1	0	0	0	3	1	1	0	0	0	1
Wolfiporia cocos	3	1	0	0	0	0	5	0	0	4	0	1	0	0	2	0

[a] Putative AAO and GOX are reported.
