# Supplementary figures and images for: Expansion of the enzymatic repertoire of the CAZy database to integrate auxiliary redox enzymes
Source: Biotechnol Biofuels. 2013 Mar 21;6:41. doi: 10.1186/1754-6834-6-41 (PMC3620520; doi:10.1186/1754-6834-6-41)

Schematic distribution of ligninolytic AA in fungal genomes

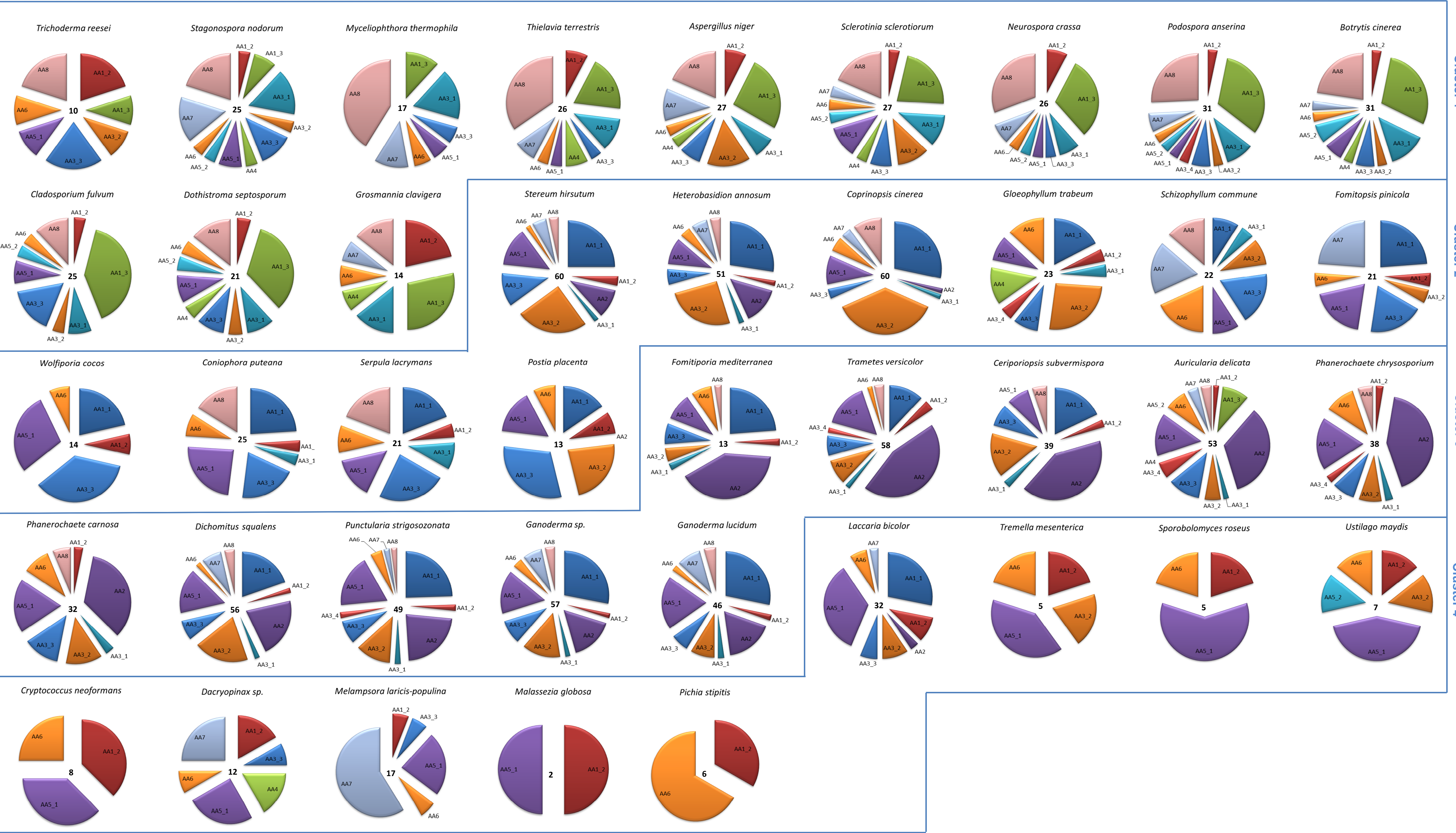

Supplement: Additional file 3: — Schematic distribution of ligninolytic AA in fungal genomes. Each genome is represented as a circle. The total number of AA is given in the middle of each circle. Groups 1 to 4 were defined according to the hierarchical clustering. (PDF 7277 kb) [file 1754-6834-6-41-S3.pdf]
